# Data concerning the Copenhagen tool: A research tool for evaluation of basic life Support educational interventions

**DOI:** 10.1016/j.dib.2020.106679

**Published:** 2020-12-31

**Authors:** Theo Walther Jensen, Andrew Lockey, Gavin D. Perkins, Anders Granholm, Kristine E. Eberhard, Asbjørn Hasselager, Thea Palsgaard Møller, Annette Kjær Ersbøll, Fredrik Folke, Anne Lippert, Doris Østergaard, Anthony J. Handley, Douglas Chamberlain, Freddy Lippert

**Affiliations:** a Copenhagen Emergency Medical Services, University of Copenhagen, Telegrafvej 5, 2750 Copenhagen, Denmark; b Danish Resuscitation Council, c/o Emergency Medical Services, Telegrafvej 5, 2750 Copenhagen, Denmark; c Emergency Department, Calderdale Royal Hospital, Halifax, United Kingdom; d Warwick Trials Unit, University of Warwick, Coventry, CV4 7AL, United Kingdom; e Department of Intensive Care, Copenhagen University Hospital – Rigshospitalet, Blegdamsvej 9, 2100 Copenhagen, Denmark; f Copenhagen Academy for Medical Education and Simulation, Capital Region of Denmark, University of Copenhagen, Copenhagen, Denmark; g National Institute of Public Health, University of Southern Denmark, Studiestræde 6, DK-1455 Copenhagen K, Denmark; h Hadstock, Cambridge, UK; i Brighton & Sussex Medical School, University of Sussex, Brighton, East Sussex, United Kingdom

**Keywords:** BLS, Education, Resuscitation, Intervention, Validation

## Abstract

The data presented in this article are supplementary data related to the research article entitled “The Copenhagen Tool: A research tool for evaluation of BLS educational interventions” (Jensen et al., 2019). We present the following supplementary materials and data: 1) a standardized scenario used to introduce the test for gathering data on internal structure and additional response process; 2) test sheets used for rating test participant via video recordings; 3) interview-guide for collecting additional response process data; 4) items deemed relevant but not essential for laypersons, first responders and health personnel in the modified Delphi consensus process; 5) inter-rater reliability values for raters using the essential items of the tool to evaluate test participants via video recordings; 6) main themes from coding interviews with raters; 7) comparison of rater results and manikin software output.

## Specifications Table

**Table:** 

Subject area	Interventions within medical education
Specific subject area	Basic life support educational interventions
Type of data	Tables and figures
How data was acquired	Standardised tests of course participants after a European Resuscitation Council (ERC) Basic Life Support (BLS) course were video recorded and presented to six raters, who rated the performance using test sheets. After evaluation, raters were interviewed about their experience using the test sheet. Interviews were transcribed and coded, and main themes are presented.
Data format	Analysed and described
Parameters for data collection	The inclusion criteria were participants who had recently passed a ERC BLS course. Three groups of participants were enlisted: laypersons, first responders and health care personnel. A standard scenario was constructed and tested.
Description of data collection	The data were collected at a post-course test where the participants were video-filmed while taking a 6-minute test of the skills acquired during a course. The test included a standard scenario and was video recorded from two angles. The recordings were rated by six experienced BLS course providers instructors from different Danish organizations representing both ERC (e.g. Danish Resuscitation Council) and non-ERC (e.g. Red Cross) CPR course providers. The instructors were provided with a list of the essential items selected during the modified Delphi consensus process specifying what should be rated as acceptable.
Data source location	Copenhagen, Denmark
Data accessibility	Original data files can be accessed by contacting corresponding the author.
Related research article	The Copenhagen Tool: A research tool for evaluation of BLS educational interventions [1].

## Value of the Data

• Development and validation of modern tools for psycho motoric tests require validation evidence from several domains. The data in this coupled article is important because it presents validation evidence from the domains not covered in the main article.
• The data in this coupled article will benefit users of the Copenhagen Tool.
• Data illustrate how the Copenhagen tool can be applied in a standard setting. This is illustrated by presenting evidence from the domain “internal structure” data from raters who rated post-course video recorded standard scenarios of ERC BLS course participants is presented.

## 1 Data Description

**Fig. 1:**
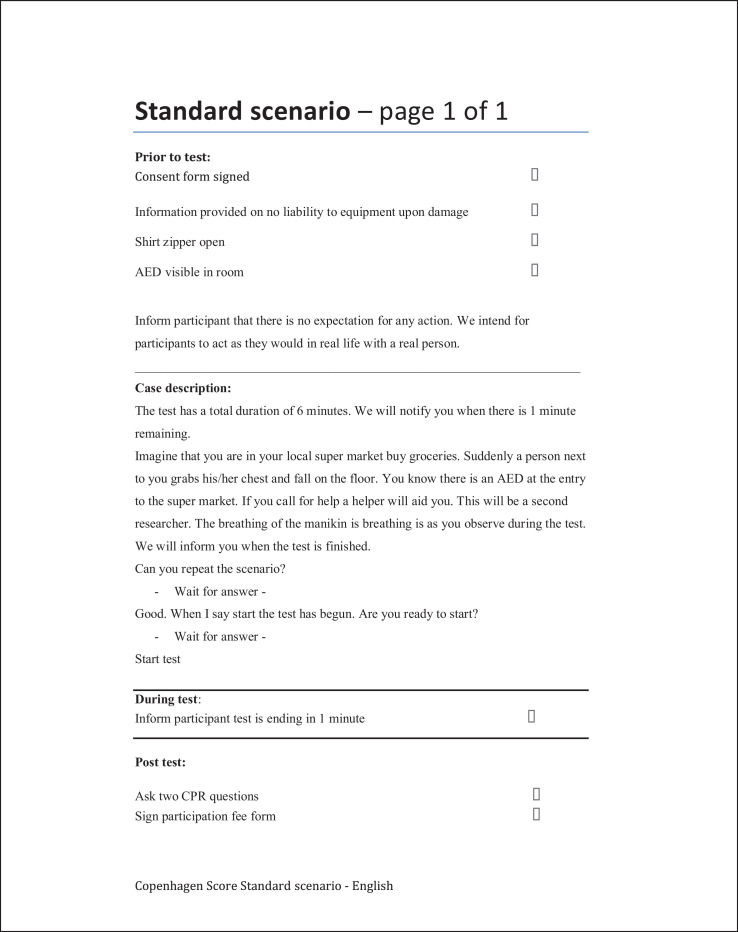
Standard scenario.

**Fig. 2:**
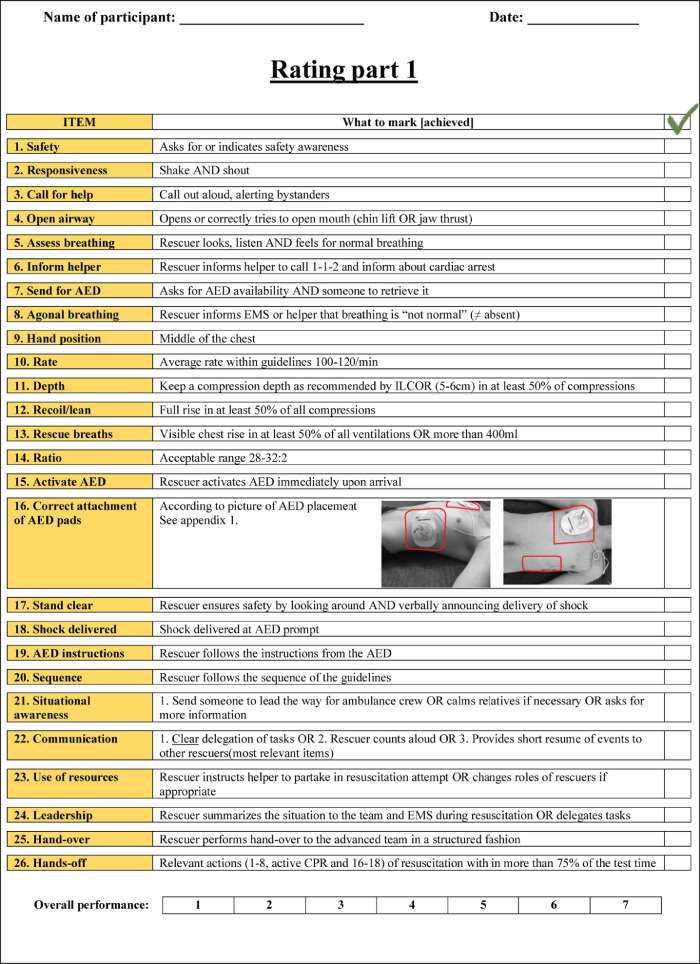

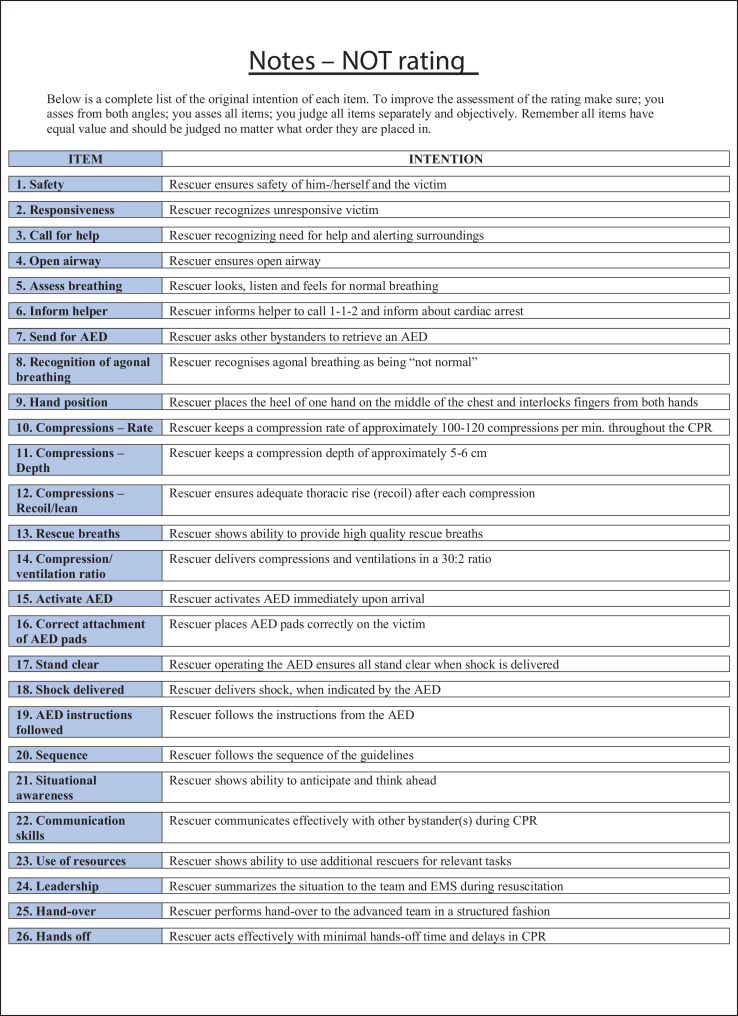

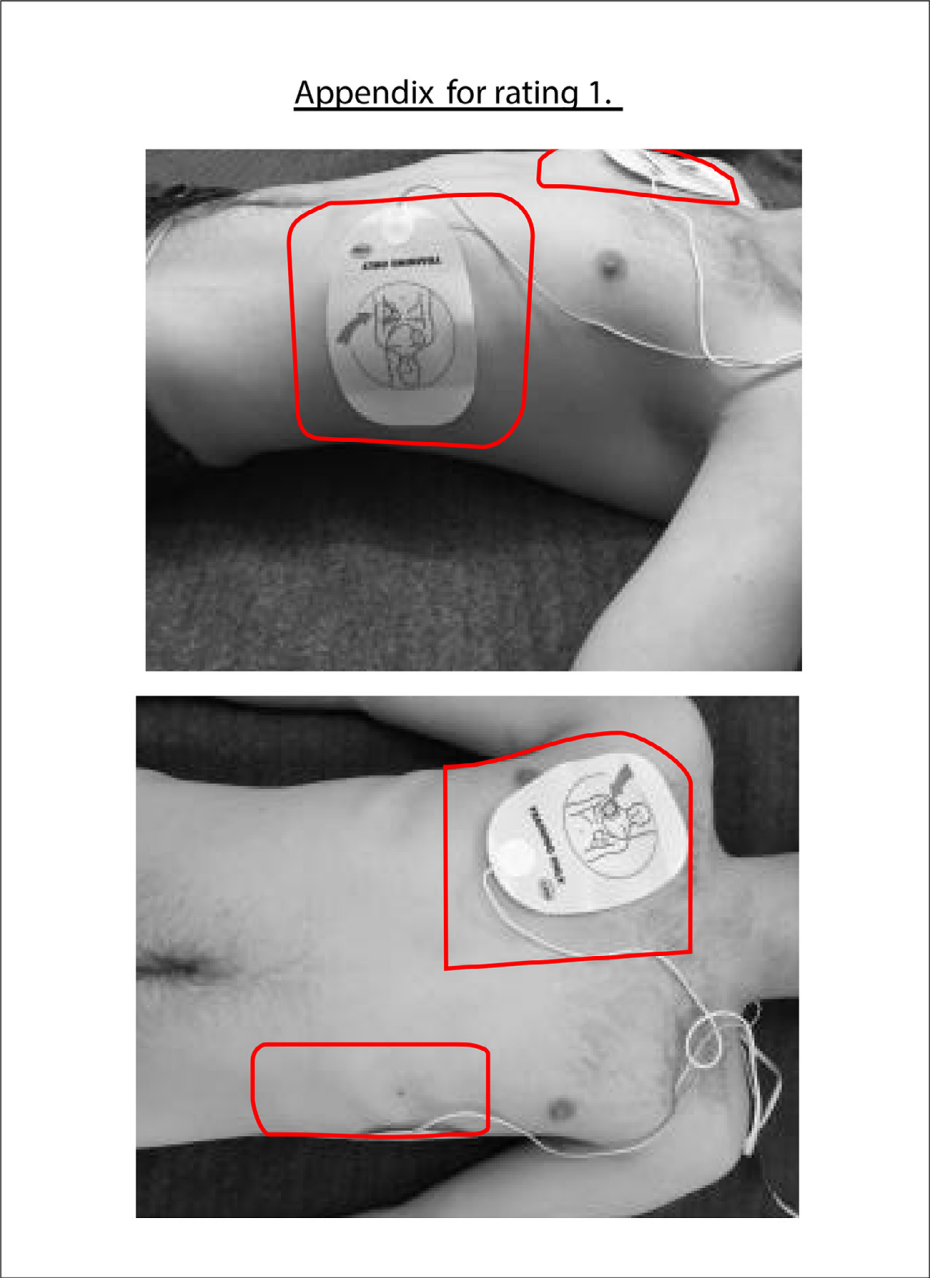

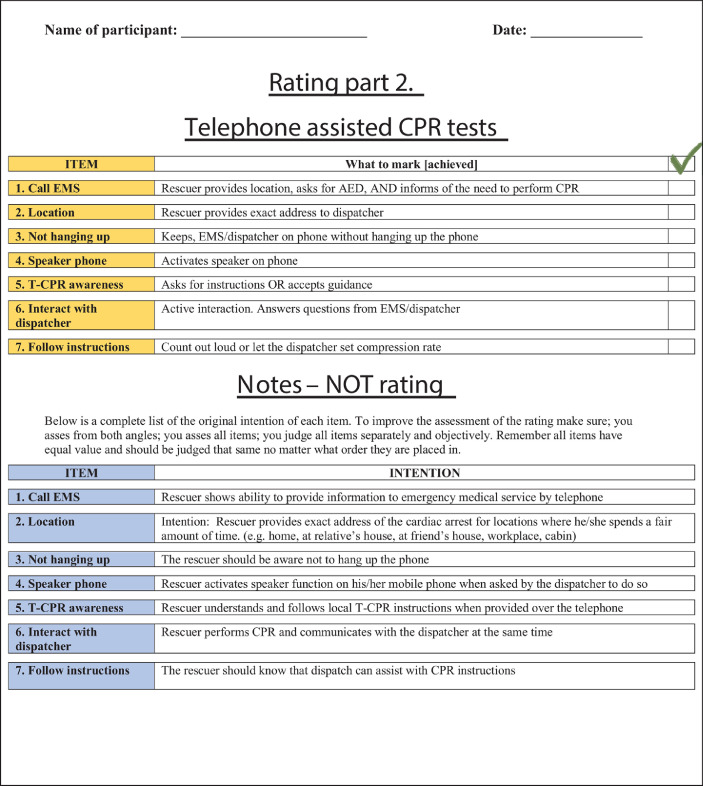
Question sheet.

**Fig. 3:**
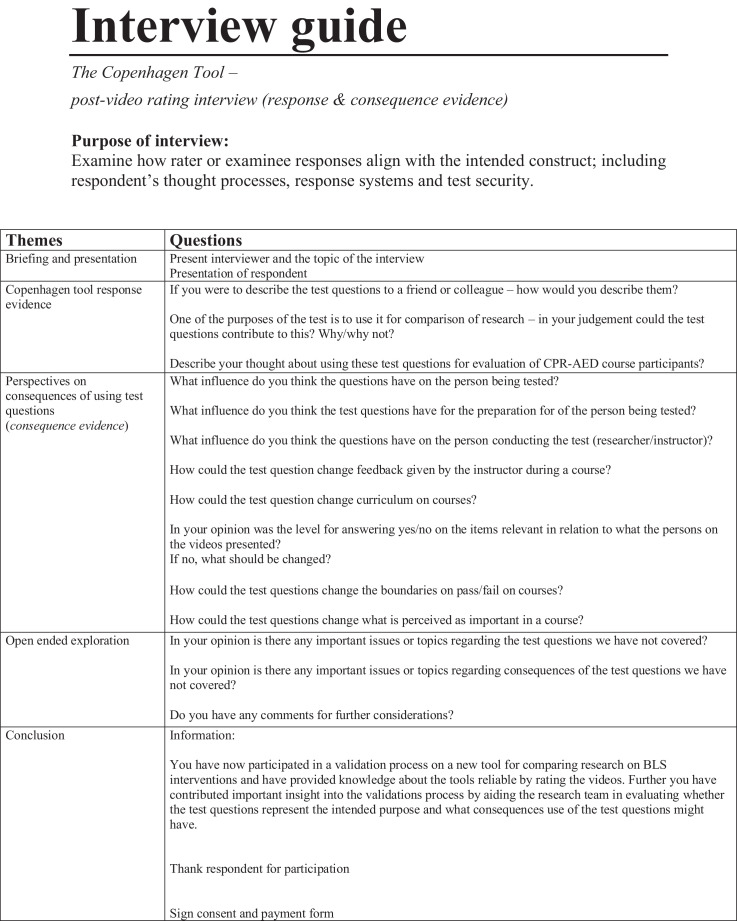
Interview guide.

The data presented in this article is supplemental data to the study “The Copenhagen Tool: A research tool for evaluation of BLS educational interventions” [1]. Fig. 1 contains the standardized scenario used to introduce the test for gathering data on internal structure and additional response process data. Fig. 2 contains question sheets used for testing internal structure evidence. Fig. 3 contains an interview-guide for collecting additional response process data. Table 1 contains all items deemed relevant but not essential for laypersons, first responders and health care personnel in the modified Delphi process. Table 2 contains internal structure evidence with Krippendorff's alpha scores, comparing the question sheet score of different raters. Table 3 contains main themes of interview coding. Table 4 contains a comparison of rater results and manikin software output. The supplementary data contains raw data, Dataset 1. Modified delphi process answers, raw data, contains all answer by experts from the modified Delphi process. The supplementary Dataset 2. Test data CPH Tool, raw data contains all answers from standardized tests.

**Table 1:** List of relevant but not essential items for each level.

Laypersons (Mr. and Mrs. Smith)		First responders (e.g. lifeguards and community first responders)		Health care personnel (e.g. doctors, nurses and EMS personnel)	
#	Item	#	Item	#	Item
1	Open airway	1	Inform helper	1	Call for help
2	DA-CPR: Follow instructions	2	Call EMS	2	Call EMS
3	DA-CPR: Speaker on	3	DA-CPR: Inform EMS	3	AED instructions followed
4	DA-CPR: Guided by dispatcher	4	DA-CPR: Follow instructions	4	Sequence
5	Compressions: Recoil/lean	5	DA-CPR: Speaker on	5	DA-CPR: Speaker on
6	Rescue breaths	6	DA-CPR: Speaker on	6	DA-CPR: Provide exact address
7	Compression/ventilation ratio	7	DA-CPR, Guided by dispatcher		
8	Activate AED	8	Sequence		
9	Stand clear	9	NTS: Situational awareness		
10	Sequence	10	NTS: Leadership		
11	NTS: Situational awareness	11	NTS: Hand-over		
12	NTS, Communication skills	12	NTS: Exchange information (address)		
13	NTS: Use of resources				
14	NTS: Leadership				
15	NTS: Hand-over				
16	NTS: Exchange information (address)				

All relevant but not essential items from the modified Delphi consensus process

AED: Automatic external defibrillator

NTS: Non-technical skills

DA-CPR: Dispatcher-assisted cardiopulmonary resuscitation

EMS: Emergency medical services

NTS: Non-Technical Skills

**Table 2:** Krippendorff's alpha scores of ratings test participant performance.

		Applicable level		
Item	Inter-rater reliability Krippendorff's alpha*, [95% CI]	Laypersons	First responders	Health care personnel
1. Safety	0.17 [−0.23, 0.44]	X	X	X
2. Responsiveness	−0.04 [−0.82, 0.67]	X	X	X
3. Call for help	0.43 [0.24, 0.61]	X	X	
4. Open airway	0.41 [0.25, 0.56]		X	X
5. Assess breathing	0.28 [0.03, 0.50]	X	X	
6. Inform helper	−0.03 [−1.0, 0.79]	X		
7. Send for AED	−0.02 [−0.5. 0.43]	X	X	X
8. Agonal breathing	0.35 [0.07, 0.61]	X	X	X
9. Hand position	0.26 [−0.2, 0.68]	X	X	X
10. Rate	−0.01 [−0.41, 0.32]	X	X	X
11. Depth	0.56 [0.33, 0.78]	X	X	X
12. Recoil/lean	0.16 [−0.09, 0.4]		X	X
13. Rescue breaths	0.34 [0.01, 0.61]		X	X
14. Ratio	0.02 [−0.50, 0.50]		X	X
15. Activate AED	0.16 [−0.14, 0.47]		X	X
16. AED instructions	0.39 [−0.52, 0.47]	X	X	
17. Correct attachment of AED pads	0.22 [−0.03, 0.47]	X	X	X
18. Stand clear	0.31 [0.01. 0.57]		X	X
19. Shock delivered	0.10 [−0.42, 0.53]	X	X	X
20. Hands-off	−0.01 [−0.2, 0.2]	X	X	X
21. Communication	0.13 [−0.5, 0.33]		X	X
22. Use of resources	0.30 [0.11, 0.49]		X	X
				
DA-CPR, Not hanging up	0.42 [0.24, 0.6]	X		
DA-CPR, Follow instructions	0.45 [0.25, 0.49]	X		

AED: Automatic external defibrillator;

NTS: Non-technical skills

DA-CPR: Dispatcher-assisted cardiopulmonary resuscitation

EMS: Emergency medical services

NTS: Non-Technical Skills

⁎ Acceptable level = 0.2

**Table 3:** Main themes of interview coding.

Response process: *Actions and thoughts of the tester*	

Research comparison	*“I think it is good to have a uniform tool [in research] (...) I would say if I am a teacher who needs to test afterwards it may also give very good insight”*
Potentials of the tool	*“For the average good instructor in Denmark, it will be a tool that is quite essential to have. And it will also be a tool that can make you focus on the teaching because you can just cut out all that nonsense of how do I think now it went? It might get cut down to some concrete evidence ... something that is measurable.”*
How to use the tool	*“When you get started with it and have run through a few slides (...)then it becomes more and more understandable(...) so it goes easier in the last one than it does in the first 2-3 ratings especially.”*

Consequence evidence: *Intended and unintended of applying the tool*	

Efforts in relation to intervention/course	*“A score or scale would help raise the lowest standard. So the low level would benefit from it (...) so basically it has a positive effect, I'm pretty sure it will (...) it will be helpful to have these things in general first aid in general.”*
Willingness to join intervention/course	*“There may also be another consequence to what is so slightly more (...) general... if taking a first aid course becomes difficult (...) Then it can have the consequence, that it causes some to say, well then they should not be in the course. I can almost hear my mother-in-law saying that if it looked (...) if it is that difficult, then you shouldn't be on course.”*
Willingness to act	*“Now let's say that the 6 points or 4 points you do not achieve when you go home from a course (...)Going home with the conviction that you are poor at first aid. I don't know if they will act in a real situation. If they were standing with cardiac arrest down the street. We hope so. "*
Objectivity in assessment and feedback	*“I think the tool can make it visible if there has been something in the teaching that has not gone through properly to the individual student. Because ... then there might be more people who have misunderstood it.. And then you have a chance (...) to clarify that.”*
Structure of intervention/course	*“You can really just take the (...) schedule and work from it. So you make sure you get it all. ”*

Themes and best illustrating citations from post rating interviews. The themes are presented in the left column. The key themes are in bold. The right column contains best illustrating citation from each theme and sub theme. = break; (. . .) = text is shortened.

**Table 4:** Rater result and manikin software output.

Item	Agreement [%]
**Hand position** *Middle of the chest*	9.3
**Rate** *Average rate within guidelines 100-120/min*	80.8
**Compression depth** *As recommended by ILCOR (5-6cm) in at least 50% of compressions*	80.8
**Recoil/lean** *Full chest rise in at least 50% of all compressions*	65.8
**Rescue breaths** *Visible chest rise in at least 50% of all ventilations OR more than 400ml*	86.7
**Ratio** *Acceptable range 28-32:2*	82.5
**Hands-off** Relevant actions of resuscitation in ≤ 75% of the test time*	60

ILCOR: The International Liaison Committee on Resuscitation

⁎ Final manikin data output was calculated by subtracting standardized flow fraction times entities when test participants performed relevant actions. The relevant actions and time entities were: ensure safety 5s; check responsiveness 5s; call for help 5s; open airway 5s; assess breathing 10s; inform helper 5s; send for AED 5s; correct attachment of AED pads 10S; stand clear 5s; and delivered shock with 5s per shock delivered.

## 2 Experimental Design, Materials and Methods

This data article includes information on tests conducted for collection of validation evidence to support the use of the Copenhagen Tool presented as the main article [1]. A total of 21 persons participated in the standardized test presented in Fig. 1. The test participants all had participated in a ERC BLS course immediately prior to the test. The tests were video-recorded and rated by six raters. Raters where experienced CPR course instructor from four different organizations operating in Denmark (Red Cross, Danish Swimming Federation, Danish First Aid Council and Danish Emergency Management Agency). Raters used the question sheet of items deemed essential for different skill levels by the expert panel, as covered in the main article [1]. A list of relevant but not essential items is presented in Table 1.

### 2.1 Response process evidence

To provide additional response process evidence, all six raters were interviewed using a semi-structured interview-guide as presented in Fig. 3. The interviews were coded using a phenomenological method modified to enable systematic condensing [2]. All minor themes from the interviews were identified by two researchers [TWJ and TPM] and condensed into subgroups of themes, and subsequently the two coders collected main themes. Main themes of the interview coding are presented in Table 3.

### 2.2 Internal structure evidence

Internal structure evidence was collected by analyzing inter-rater reliability. Raters watched the videos in separate rooms and noted achievements on a list containing all elements from all levels shown in Fig. 2. Inter-rater reliability of video ratings was assessed using Krippendorff's alpha as the reliability measure [3], [4], [5] as shown in Table 2. This reliability measure was used as it can be applied regardless of the number of raters, scale of measurements (e.g. binary and continuous), sample sizes, and presence of missing data. An alpha value of one indicates perfect agreement, while an alpha value of zero indicates complete absence of agreement. The analysis was performed using the Statistical Analysis System (version 9.4, city, country) and the KALPHA SAS macro. Krippendorff's alpha is estimated by bootstrapping using 10,000 bootstrap samples. The 95% confidence interval for Krippendorff's alpha was given as the 2.5th and 97.5th percentiles of the bootstrap distribution.

### 2.3 Relations to other variables

The evidence domain labeled “*relations to other variables*” presents associations between assessment scores and other measures of the same content. In this study evidence from this domain was collected with the scope of facilitating comparison of research in BLS educational interventions. We have compared the answers of the raters with that manikin data to provide further sources of comparison. In Table 4, the estimates of agreement between raters’ scores and manikin data output are presented.

## 3 Limitations

The authors note Fig. 2. in this paper contains image of a misplaced AED electrode pad. The long axis of the apical paddle should be orientated in a cranio-caudal direction to minimise transthoracic impedance.

## Data Accessibility

Raw data for this study is attached as supplementary materials. All data is accessible except for the video-recordings of the tested subjects. Video-recordings can be shared upon reasonable request.

## Ethics Statement

All experts agreed to participate. The Regional Ethical Committee in the Capital Region of Denmark waived the need for approval (journal number 16027743).

## Declaration of Competing Interest

The authors declare no competing interests.

## Appendix Supplementary materials

Image, application 1

Image, application 2
